# Gonadotropin-releasing hormone agonists during cyclophosphamide for ovarian protection in systemic lupus erythematosus

**DOI:** 10.1530/RAF-25-0191

**Published:** 2026-08-12

**Authors:** Mauro Francesco Pio Maiorano, Gennaro Cormio, Vera Loizzi, Brigida Anna Maiorano, Giacomo Corrado, Erica Silvestris

**Affiliations:** ^1^ Unit of Obstetrics and Gynecology, Department of Interdisciplinary Medicine (DIM), University of Bari “Aldo Moro”, Policlinico of Bari, Bari, Italy; ^2^ Gynecologic Oncology Unit, IRCCS Istituto Tumori “Giovanni Paolo II”, Bari, Italy; ^3^ Department of Translational Biomedicine and Neuroscience (DiBraiN), University of Bari “Aldo Moro”, Policlinico of Bari, Bari, Italy; ^4^ Department of Medical Oncology, IRCCS San Raffaele Hospital, Milan, Italy; ^5^ Gynecologic Oncology Unit, Department of Woman, Child and Public Health, Fondazione Policlinico Universitario A. Gemelli, IRCCS, Rome, Italy

**Keywords:** GNRH agonist therapy, GNRH-a lupus, lupus ovarian preservation, SLE ovarian protection, SLE ovarian protection systematic review, SLE ovarian protection meta-analysis, primary ovarian insufficiency, fertility preservation, cyclophosphamide adverse effects, gonadotropin-releasing hormone agonists

## Abstract

**Abstract:**

This study aimed to assess whether co-treatment with gonadotropin-releasing hormone agonists during cyclophosphamide therapy protects ovarian function and preserves fertility in women with systemic lupus erythematosus. We performed a systematic review and meta-analysis of comparative cohort studies including premenopausal women with systemic lupus erythematosus treated with intravenous cyclophosphamide with or without gonadotropin-releasing hormone agonists. The primary outcome was primary ovarian insufficiency, with pregnancy and serious adverse events as secondary outcomes. Two reviewers independently selected studies, extracted data, and assessed risk of bias. Pooled risk ratios with 95% confidence intervals were calculated using fixed-effect models. Five cohort studies including 162 women (93 with gonadotropin-releasing hormone agonists and 69 controls) met the inclusion criteria; no randomized controlled trials were available. Premature ovarian insufficiency occurred in 5 of 93 women receiving gonadotropin-releasing hormone agonists and in 28 of 69 controls, corresponding to a risk ratio of 0.16 (95% confidence interval (CI): 0.07–0.37). Pregnancy was more frequent after gonadotropin-releasing hormone agonist co-treatment (19 of 87 versus 6 of 60 women; risk ratio: 1.82, 95% CI: 0.86–3.86), although this difference did not reach statistical significance. The incidence of serious adverse events was similar between groups (10 of 36 versus 10 of 34 women; risk ratio: 0.99; 95% CI: 0.50–1.93). In premenopausal women with systemic lupus erythematosus receiving cyclophosphamide, co-treatment with gonadotropin-releasing hormone agonists markedly reduces the risk of primary ovarian insufficiency without evidence of added serious toxicity and may increase subsequent pregnancy. However, the absence of randomized controlled trials represents a major limitation, and larger prospective randomized or well-controlled studies with a standardized long-term reproductive follow-up are needed to confirm these findings.

**Lay summary:**

Systemic lupus erythematosus is an autoimmune disease that often affects young women. When the disease is severe, the drug cyclophosphamide, which can damage the ovaries and cause early menopause and infertility, may be used. With our study, we wanted to assess whether adding injections of gonadotropin-releasing hormone agonists, drugs that temporarily switch off the ovaries, can protect fertility. We combined results from five studies including 162 women with systemic lupus erythematosus who received cyclophosphamide with or without these hormone injections. Early permanent loss of ovarian function occurred in 5 of 93 women who received the injections, compared with 28 of 69 women who did not. More women in the injection group later became pregnant (19 of 87 versus 6 of 60). Serious side effects were similar in both groups. These findings suggest that temporary ovarian suppression during cyclophosphamide treatment can reduce the risk of losing fertility in young women with systemic lupus erythematosus.

## Introduction

Systemic lupus erythematosus (SLE) is a chronic autoimmune disorder characterized by periods of remission and flare, marked by multisystem inflammation that affects the skin, joints, kidneys, central nervous system, and other organs (Justiz Vaillant *et al.* 2025). The disease disproportionately affects women, with a female-to-male ratio of approximately 9:1, and its peak incidence occurs during the reproductive years. Epidemiological data estimate the incidence of SLE to be 1–10 per 100,000 person-years in the general population, mainly affecting women of reproductive age, with a prevalence of 100–200 cases per 100,000 (Soto *et al.* 2004, Jones & Giles 2016). While baseline fertility in women with SLE is often preserved, both disease-related factors, such as active inflammation and autoimmune-mediated damage, and treatment-related exposures, particularly immunosuppressive drugs, can adversely impact reproductive potential (Yan Yuen *et al.* 2008). In particular, alkylating agents, such as cyclophosphamide (CYC), a cornerstone immunosuppressive agent for severe SLE manifestations (e.g. lupus nephritis), are well known for their gonadotoxic effects leading to menstrual irregularities, ovarian reserve depletion, and premature ovarian insufficiency (POI) – historically referred to as premature ovarian failure (POF) in several older studies – in a substantial proportion of SLE patients, strongly correlating with patient age and cumulative dose (Pacucci *et al.* 2017). For example, one cohort found that up to 60% of SLE patients aged 30–40 years on cyclophosphamide developed sustained ovarian insufficiency; even patients under 30 years of age faced considerable risk (POI in <50%) (Manger *et al.* 2006). This underscores that infertility due to cyclophosphamide is a serious long-term concern, especially as more SLE patients strive to have children; thus, preventing gonadal damage has become a key aspect of SLE management. To mitigate cyclophosphamide’s gonadotoxicity, gonadotropin-releasing hormone agonists (GnRH-as) have emerged as a proactive protective strategy. Co-administering a GnRH-a and CYC induces a temporary prepubertal hormonal phase, reducing the number of maturing follicles that are exposed to cyclophosphamide’s cytotoxic effects (Manger *et al.* 2006). Despite the intuitive appeal of GnRH-a co-therapy, strong evidence of its efficacy in SLE has only begun to be collected in recent years, and scarce literature data highlight ongoing uncertainties. Many questions persist regarding the durability of ovarian protection, the ultimate impact on live birth rates, and any potential adverse events (AEs) of GnRH agonists in this context. Similarly, whether the type of GnRH agonist employed influences outcomes has not been clear. Although evidence in SLE remains lacking, the rationale for GnRH-a co-treatment is supported by randomized breast cancer trials and meta-analyses showing reduced chemotherapy-induced ovarian insufficiency and improved post-treatment pregnancy outcomes with GnRH agonists during gonadotoxic chemotherapy (Lambertini *et al.* 2018, Moore *et al.* 2019). Our meta-analysis is designed to build upon the existing evidence while addressing its limitations and filling its gaps. We aim to rigorously evaluate the effect of GnRH-a co-therapy on the prevention of POI and subsequent pregnancy rates in SLE patients receiving cyclophosphamide, while also documenting any reported AEs to address GnRH-a therapy’s safety in this particular population. Importantly, our analysis focuses specifically on SLE patients, excluding patients with malignancies, to ensure rigor, and incorporates novel subgroup analyses by age and by type of GnRH agonist, stratifications that have not been reported in previous studies. By examining outcomes across age brackets and different GnRH-a agents, we seek to determine whether efficacy or safety varies in these subpopulations. Ultimately, our introduction of age- and agent-specific analyses, combined with an up-to-date synthesis of ovarian outcomes, will help refine clinical strategies to protect fertility in women with SLE undergoing cyclophosphamide therapy.

## Materials and methods

This systematic review and meta-analysis was conducted following the Preferred Reporting Items for Systematic Reviews and Meta-Analyses (PRISMA) 2020 and Meta-analysis of Observational Studies in Epidemiology (MOOSE) guidelines (Brooke *et al.* 2021, Page *et al.* 2021). We have adhered to the relevant EQUATOR guidelines, using the Strengthening the Reporting of Observational Studies in Epidemiology (STROBE) statement for reporting observational studies (von Elm *et al.* 2007). The protocol was prospectively registered in the PROSPERO database (Registration ID: CRD420251077255). Ethical approval was not required due to the inclusion of anonymized, previously published data.

### Eligibility criteria

A systematic review was conducted according to predefined eligibility criteria, based on the Population, Intervention, Comparison, and Outcome (PICO) framework (Supplementary Table 1 (see section on Supplementary materials given at the end of the article)). We included randomized controlled trials (RCTs) and comparative cohort studies, either prospective or retrospective, that enrolled premenopausal fertile women with SLE undergoing intravenous cyclophosphamide therapy and evaluated the co-administration of GnRH-as as a strategy for fertility preservation. Eligible studies were required to include a comparator group of patients receiving cyclophosphamide without a GnRH-a and to report on at least one of the following outcomes: i) incidence of POI, including outcomes reported as POF in the original studies, ii) pregnancy rates, or iii) AEs. We excluded case reports, case series, narrative reviews, systematic reviews that did not report original data, editorials, conference abstracts lacking full peer-reviewed publication, and non-comparative studies. Additionally, studies not published in English or those involving populations with malignancies or non-SLE autoimmune diseases were excluded. Only studies with sufficient and extractable outcome data were included in the quantitative synthesis.

### Search strategy

A comprehensive literature search was conducted using PubMed, Scopus, and Web of Science databases from inception (January 1, 2000) to May 31, 2025. The following Boolean search terms were used: (“GnRH agonist” OR “gonadotropin-releasing hormone analogue” OR “leuprolide” OR “triptorelin” OR “decapeptyl”) AND (“systemic lupus erythematosus” OR “SLE”) AND (“cyclophosphamide”) AND (“fertility” OR “ovarian failure” OR “ovarian insufficiency” OR “pregnancy” OR “gonadotoxicity”). The complete, reproducible line-by-line strategies used in the final search are reported in Supplementary File 1. All records were exported with full citation data and abstracts, merged, and de-duplicated before screening (EndNote/Rayyan acceptable). No language restriction was applied initially. After removing duplicates, titles and abstracts were independently screened by two reviewers (MFPM and ES). A full-text assessment was conducted based on predefined inclusion criteria. Disagreements were resolved by consensus. Additionally, reference lists of relevant systematic reviews and included articles were screened manually to identify further eligible studies.

### Study selection

Two reviewers (MFPM and BAM) independently screened titles and abstracts retrieved from the initial search. Full-text articles were evaluated for eligibility based on the inclusion and exclusion criteria. Any disagreements were resolved by discussion and, if necessary, consultation with a third reviewer (ES). The study selection process followed the PRISMA guidelines, and a PRISMA flowchart (Supplementary Fig. 1) was constructed to document the selection process.

### Data extraction

Data were independently extracted by two reviewers (MFPM and BAM) using a standardized data collection form. Extracted data included study design, number and demographic characteristics of participants, type and dosage of GnRH-a, cumulative cyclophosphamide dose, duration of follow-up, and reported outcomes: POI, pregnancy rate, and AEs. Where data were incomplete or unclear, the corresponding authors were contacted for clarification.

### Risk-of-bias assessment

Risk of bias was independently assessed by two authors using the Risk Of Bias In Non-randomized Studies – of Interventions (ROBINS-I) tool, appropriate for evaluating observational studies (Sterne *et al.* 2016). Seven domains were evaluated: bias due to confounding, selection of participants, classification of interventions, deviations from intended interventions, missing data, measurement of outcomes, and selection of reported results. Each study was rated as having low, moderate, or serious risk in each domain. Discrepancies were resolved through discussion.

### Certainty of evidence

We assessed the certainty of evidence using the GRADE approach across five domains: risk of bias, inconsistency, indirectness, imprecision, and publication bias (Schünemann *et al.* 2023). Outcomes prioritized were i) POI, ii) pregnancy, and iii) serious AEs.

### Data synthesis and statistical analysis

The meta-analysis was performed using R software (version 4.4.2) with the meta and metafor packages. Dichotomous outcomes were analyzed using risk ratios (RRs) with 95% confidence intervals (CIs). A fixed-effect model was used in the absence of heterogeneity (*I*^2^ < 50%, *P* > 0.10), while a random-effects model was applied otherwise. Heterogeneity was assessed using Cochran’s Q test and the *I*^2^ statistic (Cochran 1950). Publication bias was evaluated through funnel plots and visual inspection of symmetry. Subgroup analyses were performed based on age-group (≤25 vs 26–30 years) and type of GnRH agonist (leuprolide and triptorelin). Statistical significance was set at a two-sided *P*-value of <0.05.

## Results

### Study selection and characteristics

Initially, a total of 127 studies were identified through comprehensive searches of electronic databases, including PubMed, Embase, Scopus, and Web of Science, using predefined keywords related to GnRH agonists, systemic lupus erythematosus, cyclophosphamide, and fertility preservation. After removing 42 duplicates, 85 records remained for title and abstract screening. Of these, 62 articles were excluded due to irrelevance to the research question, such as reviews, editorials, animal studies, or studies not involving GnRH-a use during cyclophosphamide therapy. The full texts of the remaining 23 articles were assessed for eligibility. Following the full-text review, 18 studies were excluded for reasons including lack of a control group, insufficient outcome data, or overlapping populations. Supplementary Fig. 1 presents the PRISMA flowchart for study selection. Ultimately, five comparative cohort studies (three retrospective and two prospective) met all the prespecified inclusion criteria and were included in the systematic review and meta-analysis (Blumenfeld *et al.* 2000, 2011, Somers *et al.* 2005, Koga *et al.* 2018, Orefice *et al.* 2020). No RCTs fulfilling the eligibility criteria were identified. Table 1 summarizes the included studies and their main findings. The studies were conducted across diverse geographical settings and involved a combination of prospective and retrospective cohort designs. The mean age of the participants across all studies was 26.6 years (range: 16–43 years). Most patients presented with moderate-to-severe SLE, primarily characterized by lupus nephritis or central nervous system (CNS) involvement. Cyclophosphamide exposure, when reported, ranged approximately from 3 to 33.5 g, although cumulative dose data were not uniformly available across all included studies. A total of 93 patients received a GnRH-a as a fertility preservation strategy, with the most commonly used agent being triptorelin. The control group consisted of 69 patients who did not receive a GnRH-a. All included studies administered GnRH-a before or concurrent with cyclophosphamide initiation, with most specifying initiation at least 10 days before CYC to mitigate risk from the transient estrogen surge. While no study directly compared timing strategies, consistent timing practices across studies reporting reduced POI support early administration before gonadotoxic exposure as best practice. Table 2 summarizes the patients’ characteristics and the overall population’s findings.

**Table 1:** Summary of the included studies.

Study	Design	Total, *n*	Intervention		Comparator (CYC alone)		POI rate (*n*)	Pregnancy rate (*n*)	Any grade AEs
			*n*	Dose (3.75 mg IM q1m)	*n*	mCD			
Koga *et al.* (2018)	RS	30	16	Leuprolide	14	5.0 g	6% vs 50% (1 vs 7)	6.25% vs 0% (1 vs 0)	1/16 vs 1/14
Orefice *et al.* (2020)	RS	33	18	Triptorelin	15	NA	11% vs 33% (2 vs 5)	16.7% vs 0% (3 vs 0)	NA
Somers *et al.* (2005)	PS	40	20	Leuprolide	20	12.9 g (1 case 33.5 g)	5% vs 30% (1 vs 6)	35% vs 15% (7 vs 3)	9/20 vs 9/20
Blumenfeld *et al.* (2000)	PS	15	6	Triptorelin (Decapeptyl)	9	NA	0% vs 55.6% (0 vs 5)	NA	NA
Blumenfeld *et al.* (2011)	RS	44	33	Triptorelin (Decapeptyl)	11	9.9 g (3–25)	3% vs 45.5% (1 vs 5)	21% vs 27.2% (8 vs 3)	NA

AE(s), adverse event(s); CYC, cyclophosphamide; IM, intramuscular; mCD, mean cumulative dose; NA, not available; q1m, every month; POI, premature ovarian insufficiency; RS, retrospective study; PS, prospective study.

**Table 2:** Univariate logistic regression analysis of factors associated with poor FertiQoL (score <62) among infertile men in Henan province (*n* = 319).

Variable	OR	95% CI	*P* value
Monthly income (low vs high)	1.357	1.018–1.809	0.037
Infertility duration (per year increase)	2.133	1.489–3.056	<0.001
Severe SD (ASEX score ≥ 19, yes vs no)	3.629	2.127–6.192	<0.001
Age (per year increase)	1.338	0.988–1.812	0.059
Education level (primary/secondary vs tertiary)	1.071	0.79–1.452	0.66
ART failure (yes vs no)	1.427	0.863–2.361	0.166

OR, crude odds ratio; 95% CI, 95% confidence interval.

Poor FertiQoL was defined as an overall FertiQoL score <62. For continuous variables (age and infertility duration), OR represents the change in odds per unit increase. For categorical variables, reference categories are indicated in parentheses. Variables with *P* < 0.25 were selected as candidates for multivariable analysis.

### Primary outcome: premature ovarian insufficiency

The incidence of POI was significantly lower among patients treated with a GnRH-a compared to controls in all studies reporting this outcome. Pooled data revealed a POI rate of approximately 5.3% (5/93) in the GnRH-a group versus 40.5% (28/69) in the control group. The most significant differences were reported by Orefice *et al.* (2020) (*P* = 0.0002), Koga *et al.* (2018) (*P* = 0.013), and Somers *et al.* (2005) (*P* < 0.05).

### Risk-of-bias assessment results

To contextualize the primary outcome findings, the risk of bias for the five included studies was assessed using the ROBINS-I tool (Sterne *et al.* 2016). Three studies were rated as having an overall low risk of bias (Blumenfeld *et al.* 2000, Somers *et al.* 2005, Koga *et al.* 2018). These studies demonstrated appropriate control for confounding, clear classification of interventions, minimal deviations from intended treatments, consistent outcome assessment, and low attrition. The remaining two studies had a moderate overall risk of bias, largely due to their retrospective design, moderate concerns in participant selection and intervention classification, and incomplete adjustment for confounders (Blumenfeld *et al.* 2011, Orefice *et al.* 2020). Nevertheless, all included studies used standardized definitions of POI and applied consistent hormonal and clinical monitoring, leading to a low risk of detection bias across the board. No study exhibited serious concerns related to missing data or selective outcome reporting. Overall, the methodological quality of the included studies supports the robustness of the pooled estimates, while also highlighting the need for future prospective trials (Supplementary Table 2).

### Secondary outcomes: pregnancy rates and adverse events

Successful pregnancies were reported more frequently in the GnRH-a-treated groups across most studies (Blumenfeld *et al.* 2000, Somers *et al.* 2005, Koga *et al.* 2018, Orefice *et al.* 2020). Specifically, pregnancy rates in the GnRH-a arms ranged from 6.25 to 35%, compared to 0–27.2% in the control groups. While some studies reported no pregnancies in the control arm, one study observed a slightly higher pregnancy rate in the control group (27.2 vs 21%) (Blumenfeld *et al.* 2011). Overall, these findings suggest a trend toward higher reproductive success with GnRH-a administration, although results were variable across studies. No new or unexpected safety concerns occurred between subgroups, with the most common AEs being osteoporosis, abnormal uterine bleeding, and depression.

### Meta-analysis

**Figure 1:**
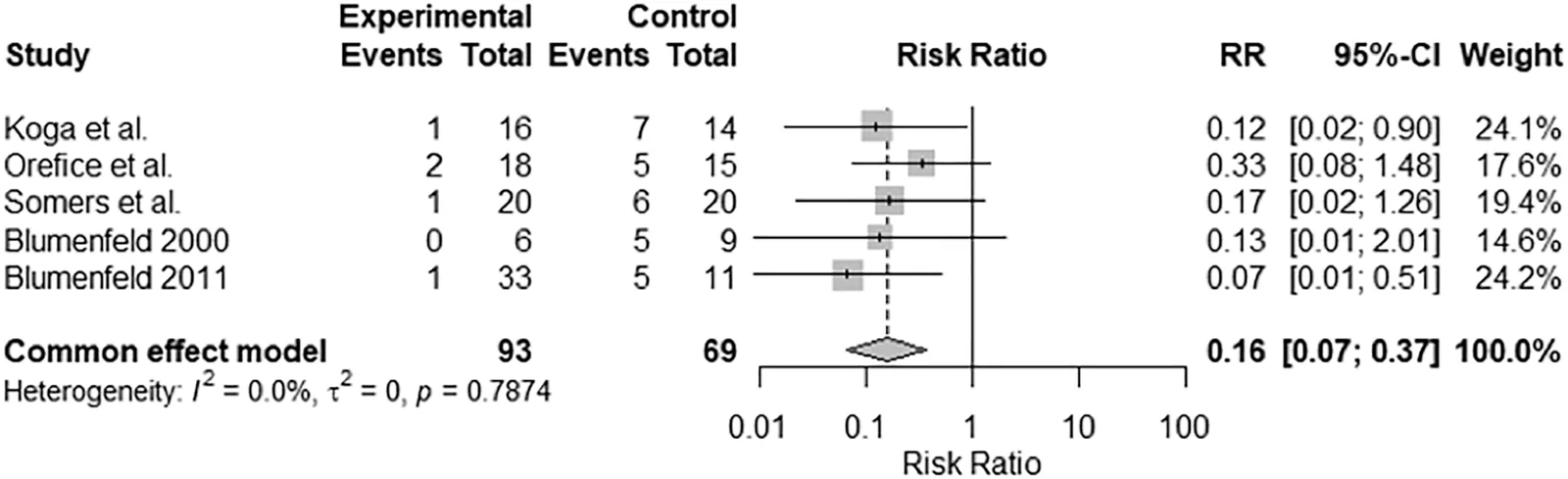
GnRH agonists reduce POI during cyclophosphamide. Study-level and pooled risk ratios (log scale) for premature ovarian insufficiency comparing GnRH-a + CYC vs CYC alone across five cohorts. The diamond shows the common-effect estimate RR = 0.16 (95% CI: 0.07–0.31) with *I*^2^ = 0%, indicating a consistent, marked reduction in POI favoring GnRH-a.

**Figure 2:**
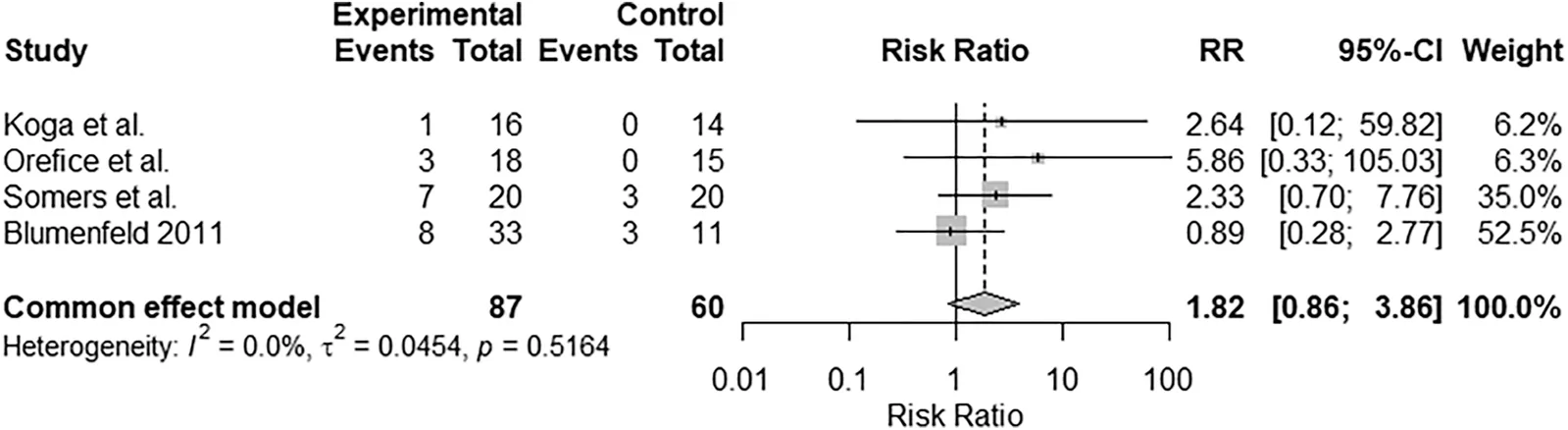
Pregnancy after cyclophosphamide with GnRH agonist co-therapy. Study-level and pooled risk ratios (log scale) for posttreatment pregnancy comparing GnRH-a + CYC vs CYC alone across four cohorts. The diamond shows the common-effect estimate RR = 1.82 (95% CI: 0.86–3.86) with *I*^2^ = 0%, indicating a consistent trend favoring GnRH-a, though not statistically significant.

**Figure 3:**
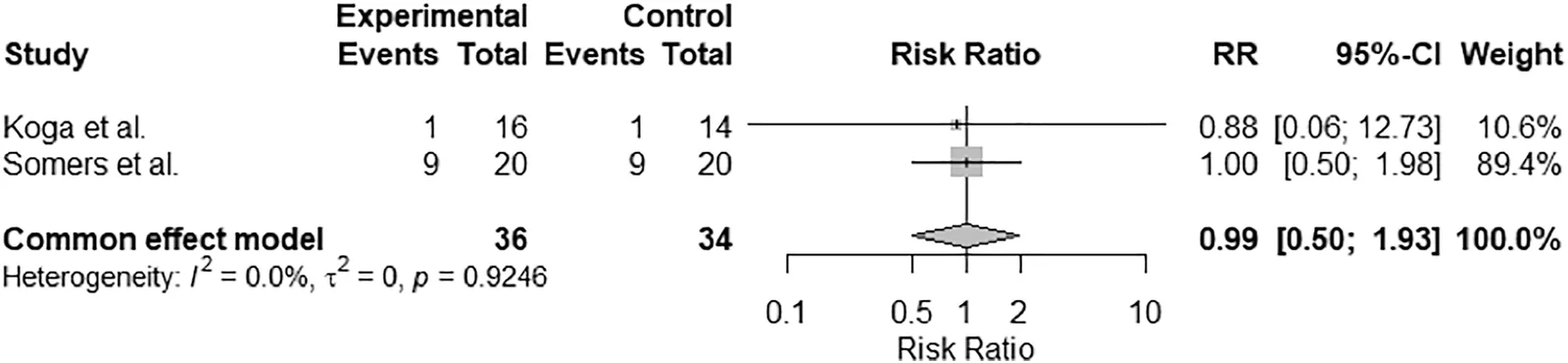
Serious adverse events with GnRH agonist co-therapy. Study-level and pooled risk ratios (log scale) for serious AEs comparing GnRH-a + cyclophosphamide vs cyclophosphamide alone across two cohorts. The diamond shows the common-effect estimate RR = 0.99 (95% CI: 0.50–1.93; *I*^2^ = 0%), indicating no clear difference in serious adverse events between groups.

The pooled effect estimate, calculated using a fixed-effect model, demonstrated a significant reduction in the risk of POI among patients treated with GnRH-a compared to controls, with an RR of 0.16 (95% CI: 0.07–0.37) (Blumenfeld *et al.* 2000, 2011, Somers *et al.* 2005, Koga *et al.* 2018, Orefice *et al.* 2020). Heterogeneity among the studies was negligible (*I*^2^ = 0%, *P* = 0.7874), demonstrating consistent findings across all included analyses. The individual study estimates ranged from an RR of 0.07 to 0.33, all favoring the GnRH-a group, suggesting a strong and homogeneous protective effect of GnRH-as against ovarian insufficiency induced by cyclophosphamide in SLE patients (Fig. 1). The funnel plot for the meta-analysis included five studies (Blumenfeld *et al.* 2000, 2011, Somers *et al.* 2005, Koga *et al.* 2018, Orefice *et al.* 2020). The studies appear symmetrically distributed around the pooled effect size (RR = 0.16), with risk ratio values ranging approximately from 0.07 to 0.33 and standard errors concentrated in the lower portion of the plot. The symmetry of the plot suggests an absence of small-study effects and no apparent evidence of publication bias (Supplementary Fig. 2). The pregnancy rate meta-analysis included four studies, comprising 87 patients in the GnRH-a group and 60 in the control group (Somers *et al.* 2005, Blumenfeld *et al.* 2011, Koga *et al.* 2018, Orefice *et al.* 2020). The RR, calculated using a fixed-effect model, was 1.82 (95% CI: 0.86–3.86), suggesting a higher likelihood of pregnancy among patients receiving a GnRH-a. Heterogeneity was low (*I*^2^ = 0%, *P* = 0.5164), indicating consistency across the included studies. Individual study estimates ranged from an RR of 0.89 to 5.86, with three out of four studies showing results in favor of the GnRH-a group (Fig. 2). The pooled analysis of serious AEs, comparing those who received a GnRH-a versus those who did not, included two studies, with a total of 36 patients in the GnRH-a group and 34 in the control group (Somers *et al.* 2005, Koga *et al.* 2018). The pooled RR for serious AEs was 0.99 (95% CI: 0.50–1.93), suggesting no statistically significant difference in the incidence of serious AEs between the two groups. There was no evidence of heterogeneity (*I*^2^ = 0%, *P* = 0.9246), showing consistency in the effect estimate across studies (Fig. 3).

### Subgroup analyses

**Figure 4:**
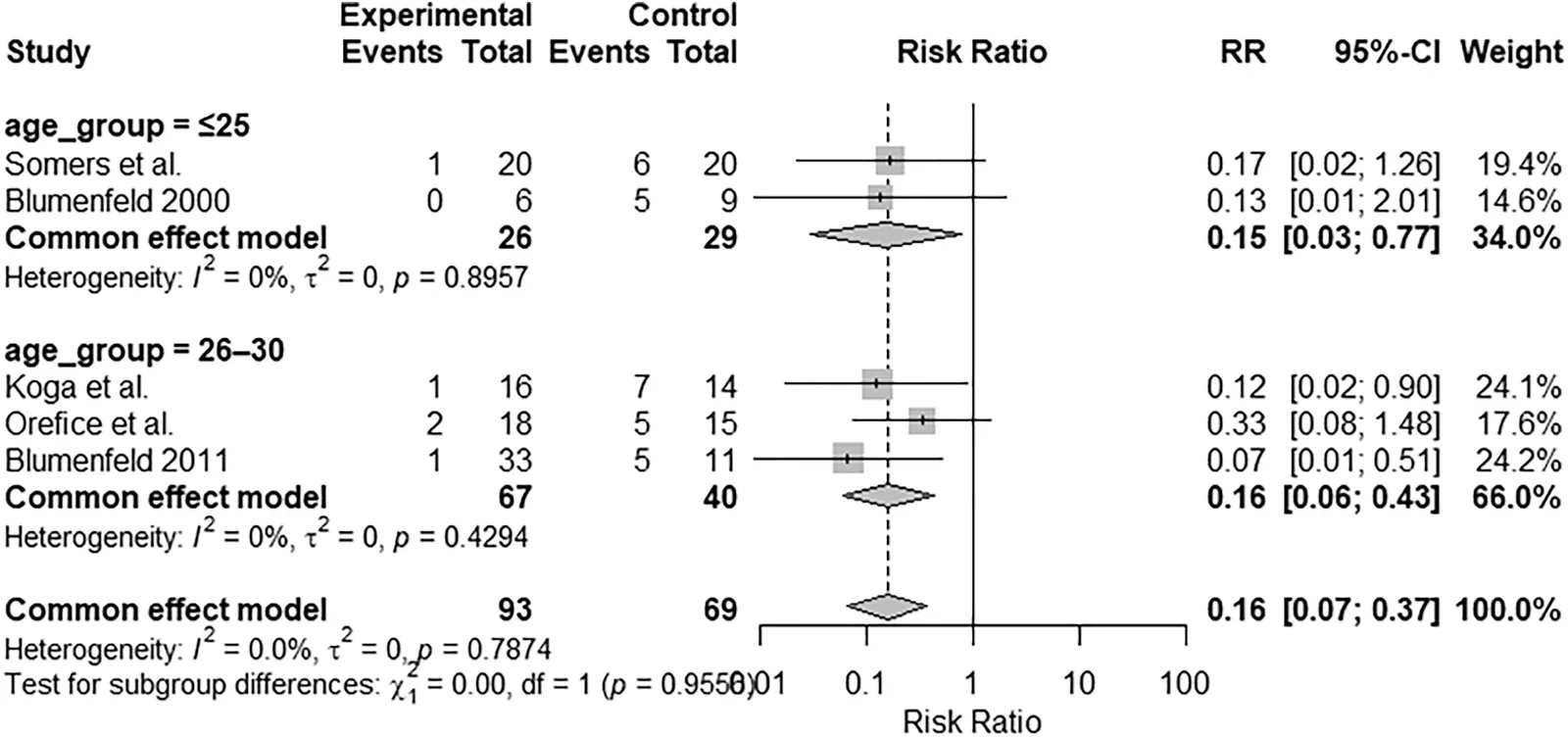
Age-stratified effect on POI (≤25 vs 26–30 years). Subgroup meta-analysis of premature ovarian insufficiency comparing GnRH agonist + cyclophosphamide vs cyclophosphamide alone. Pooled RR for ≤25 years: 0.15 (95% CI: 0.03–0.77; *I*^2^ = 0%); pooled RR for 26–30 years: 0.16 (95% CI: 0.07–0.37; *I*^2^ = 0%). Overall common-effect RR = 0.16 (95% CI: 0.07–0.37). No evidence of subgroup differences (*χ*^2^ ≈ 0.00; *P* ≈ 0.96).

**Figure 5:**
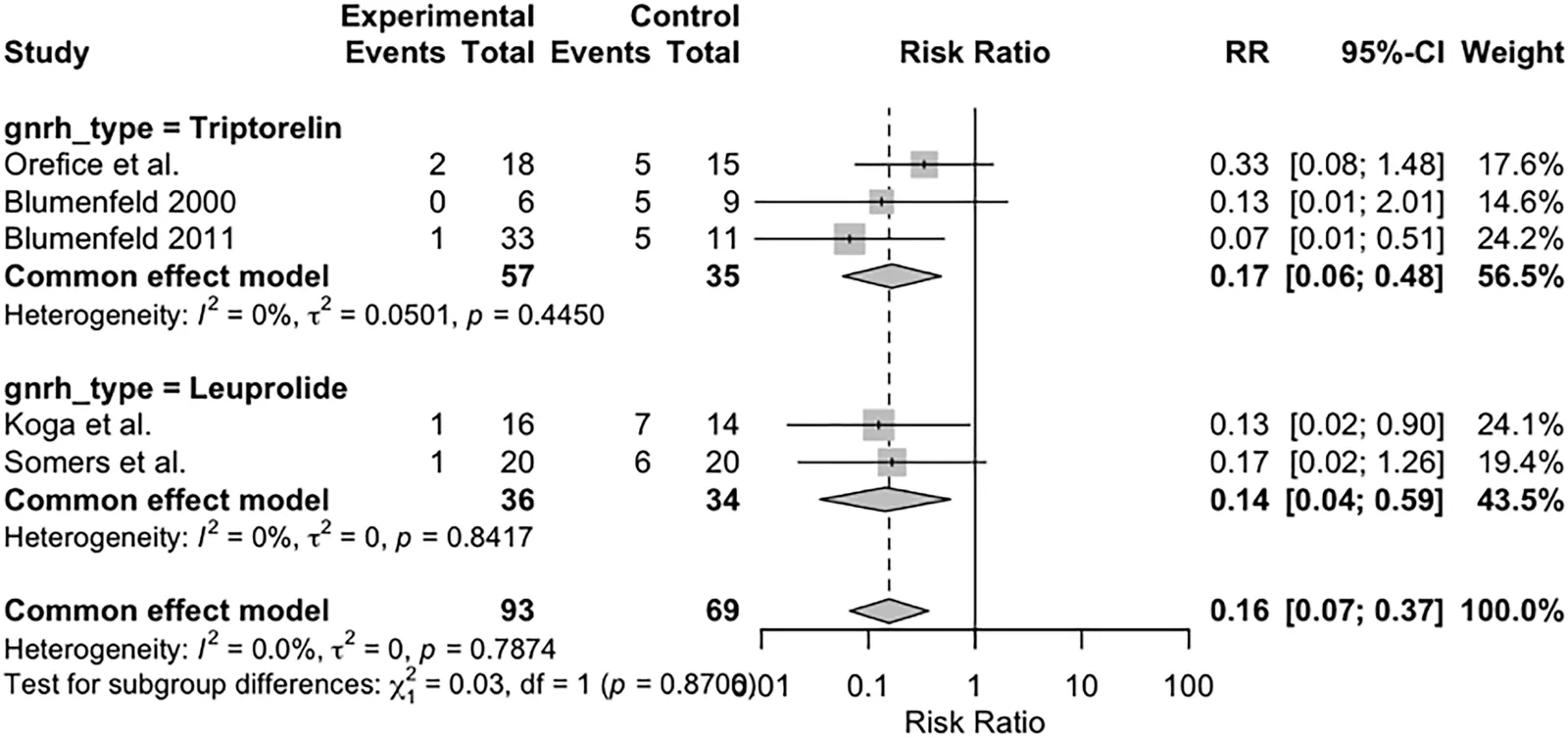
POI reduction by GnRH agonist type (triptorelin vs leuprolide). Subgroup meta-analysis comparing GnRH-a + cyclophosphamide vs cyclophosphamide alone by agent. Triptorelin: pooled RR = 0.17 (95% CI: 0.06–0.48; *I*^2^ = 0%). Leuprolide: pooled RR = 0.13 (95% CI: 0.02–0.94; *I*^2^ = 0%). Overall common-effect RR = 0.16 (95% CI: 0.07–0.37) with no subgroup difference (*χ*^2^ = 0.03; *P* = 0.87).

The subgroup meta-analysis of POI according to patient age (≤25 vs 26–30 years) included all the studies, involving a total of 93 patients in the GnRH-a groups and 69 in the control groups (Blumenfeld *et al.* 2000, 2011, Somers *et al.* 2005, Koga *et al.* 2018, Orefice *et al.* 2020). In patients aged ≤ 25 years, the pooled RR for POI was 0.15 (95% CI: 0.03–0.77), showing a significantly reduced risk in those treated with a GnRH-a. In the age-group of 26–30 years, the pooled RR was 0.16 (95% CI: 0.06–0.43), also confirming a protective effect. No heterogeneity was observed within or between subgroups (*I*^2^ = 0% for all comparisons), and the test for subgroup differences was not statistically significant (*χ*^2^ = 0.00, *P* = 0.9558), suggesting that the protective effect of GnRH-as was consistent across age strata (Fig. 4). The second subgroup analysis evaluated the impact of different GnRH-a types (triptorelin and leuprolide) on the prevention of POI in patients with SLE treated with cyclophosphamide. The triptorelin subgroup, based on data from three studies, yielded a pooled RR of 0.17 (95% CI: 0.06–0.48), demonstrating a statistically significant reduction in the risk of ovarian insufficiency (Blumenfeld *et al.* 2000, 2011, Orefice *et al.* 2020). The leuprolide subgroup included two studies, with a pooled RR of 0.14 (95% CI: 0.04–0.59), also indicating a significant protective effect. There was no detectable heterogeneity within either subgroup (*I*^2^ = 0% for both) (Somers *et al.* 2005, Koga *et al.* 2018). The overall effect across all studies remained consistent, with a combined RR of 0.16 (95% CI: 0.07–0.37). The test for subgroup differences showed no statistically significant variation in effect between GnRH-a formulations (*χ*^2^ = 0.03, df = 1, *P* = 0.8706) (Fig. 5).

### Certainty of evidence assessment

There were five comparative cohorts (*n* = 162; 93 GnRH-a patients and 69 controls). Pooled effects are as follows: POI: RR of 0.16 (95% CI: 0.07–0.37); pregnancy: RR of 1.82 (95% CI: 0.86–3.86); and serious AEs: RR of 0.99 (95% CI: 0.50–1.93). POI events were 5/93 vs 28/69 across studies; pregnancies were 19/87 vs 6/60 in the four studies reporting this outcome; and serious AEs were 10/36 vs 10/34 in two studies. A summary of findings is presented in Supplementary Table 3.

## Discussion

### Fertility outcomes: premature ovarian insufficiency and pregnancy

Our systematic review and meta-analysis confirm that a co-treatment combining a GnRH-a with a chemotherapy agent produces an appropriate reduction in POI risk among women with SLE undergoing cyclophosphamide therapy. We found an ≈84% relative risk reduction in POI with GnRH-as, an effect both statistically significant and consistent across studies. Without ovarian protection, up to 50% of young SLE patients on cyclophosphamide and nearly 100% of those aged over 30 years have been reported to develop sustained POI (Blumenfeld *et al.* 2000). In the study by Blumenfeld *et al.* (2000), none of the eight GnRH-a-treated patients suffered POI, versus over half (five out of nine) of the untreated controls. Likewise, the study by Somers *et al.* (2005) observed POI in only 5% of GnRH-a-treated women compared to 30% of matched controls. These convergent findings underscore that GnRH-a co-therapy can largely preserve ovarian function in lupus disease. Our results align with the existing literature: a recent meta-analysis that included rheumatic disease patients reported significantly higher odds of ovarian preservation with GnRH-as (Ejaz *et al.* 2022). In fact, European guidelines now recognize GnRH-as as the most effective strategy to prevent cyclophosphamide-induced gonadotoxicity in SLE, noting an 88% reduction in POI risk (Fanouriakis *et al.* 2019). In addition to preserving menses, GnRH-as may improve the likelihood of future pregnancy. In our analysis, the pregnancy rate was higher in the GnRH-a group (RR: ∼1.8, an ∼82% relative increase). The trend supporting GnRH-as is nonetheless consistent with the biological expectation and prior findings (Ejaz *et al.* 2022). Importantly, emerging long-term follow-up data support a real fertility benefit. For example, in the eight-year cohort study by Orefice *et al.* (2020), only the GnRH-a-co-treated patients achieved successful pregnancies, whereas none of the cyclophosphamide-only patients did. This suggests that ovarian function preserved by GnRH-as can translate into actual fertility outcomes when patients later attempt conception. Admittedly, not all women in these studies actively pursued pregnancy, and factors such as disease activity, damage, and antiphospholipid syndrome also impact fertility. Thus, as maintaining ovarian function is a prerequisite for natural fertility, our findings provide a strong rationale for GnRH-a use in any young women with SLE who might desire future pregnancy (Ejaz *et al.* 2022).

### Safety outcomes

The addition of GnRH-as did not increase the risk of mild-to-serious AEs in our analysis. The pooled risk of mild-to-severe AEs (e.g. hospitalization-requiring complications or lupus flares) was neutral when comparing GnRH-a versus no GnRH-a, with no statistically significant difference between groups. All studies reported that GnRH-as were generally well tolerated, and we observed no heterogeneity in AE outcomes across trials. This safety profile is consistent with prior experience and the known pharmacology of GnRH-as (Tarlatzis & Bili 2004). The expected side effects of GnRH-as (hypoestrogenic symptoms such as hot flashes, mood changes, or minor bone density loss) are transient and were not reported in the SLE cohorts. Notably, there was no signal of increased SLE disease flares attributable to GnRH-a-induced hormonal changes, an important consideration for patients with rheumatic disease. Overall, our findings reinforce that GnRH-a co-therapy confers a favorable risk–benefit balance: it dramatically lowers POI risk without introducing significant safety concerns. Clinicians can thus be confident that protecting the ovaries with GnRH analogues does not compromise patient safety or lupus control, allowing broad implementation of this intervention in eligible patients.

### Age and GnRH agonist type

A unique contribution of this meta-analysis is the stratified analyses exploring whether GnRH-a efficacy varies by patient age or by the specific GnRH agonist used. By age-group, we found that the protective effect of GnRH-as was maintained in both younger and older reproductive-age women. Patients aged ≤ 25 years derived a significant benefit, and those in the age-group of 26–30 years had a virtually identical risk reduction, with no significant difference between age strata. In other words, GnRH-as were highly effective across the board in women from their late teens through their late twenties. This finding is clinically important given the strong influence of age on cyclophosphamide-induced ovarian insufficiency risk. Older patients have higher baseline susceptibility to POI, yet our results indicate that they still gain as much relative protection from GnRH-as as younger patients do. Thus, GnRH-a co-therapy should be offered not only to very young patients but also to those in their late twenties (and presumably beyond, if still premenopausal), as the benefit does not appear to diminish in this older subset. Prior studies lacked the power or focus to address this age question, so our analysis provides new evidence that age should not be a barrier to using GnRH agonists for ovarian preservation in SLE. Subgroup meta-analysis by GnRH agonist type demonstrates equivalent ovarian protection with both agents. This novel finding suggests the ovarian-protective effect is likely a class effect of GnRH-as. For clinicians, this means that the choice of GnRH-a can be guided by practical factors, such as availability, cost, or familiarity, rather than efficacy concerns. Previous lupus studies did not differentiate; thus, our analysis fills that gap, providing evidence that either GnRH agonist option is a valid and effective choice for fertility preservation.

### Comparison with the existing literature

Converging evidence from guidelines and oncology trials supports our conclusions. The American College of Rheumatology (ACR) 2020 reproductive health guideline conditionally recommends monthly GnRH agonist co-therapy for premenopausal patients receiving monthly IV cyclophosphamide, explicitly noting age and cumulative dose dependence and that the evidence, while positive, is limited (Sammaritano *et al.* 2020). In oncology, the breast cancer literature provides a stronger randomized evidence base for this strategy. A meta-analysis of RCTs in early breast cancer (five RCTs; *n* = 873) showed that a GnRH-a during chemotherapy halves the odds of chemotherapy-induced POI and increases post-treatment pregnancies without compromising survival, reinforcing a class effect that plausibly extends to gonadotoxic alkylators used in SLE (Lambertini *et al.* 2018). Similarly, the randomized POEMS/S0230 trial demonstrated higher pregnancy incidence and lower ovarian insufficiency with goserelin added to cyclophosphamide-containing regimens, with no adverse impact on disease outcomes, encouraging biologic and clinical plausibility for benefit in non-oncologic cyclophosphamide exposure (Moore *et al.* 2019). Reflecting this cross-disciplinary evidence, ASCO’s most recent guideline update (2025) advises that GnRH-as should not replace established fertility preservation methods but may be offered as an adjunct therapy and for menstrual suppression when urgent therapy is needed, aligned with our interpretation that GnRH-as serve as a protective co-therapy rather than a stand-alone FP strategy (Su *et al.* 2025). Finally, foundational SLE cohorts quantifying cyclophosphamide gonadotoxicity, most notably Mok *et al.* (1998), which identified age at exposure and cumulative dose as dominant predictors, provide the risk framework that justifies ovarian protection in lupus even before disease-specific GnRH-a data are available.

### Strengths and limitations

This meta-analysis was conducted with a focused and rigorous methodology to ensure high relevance to SLE clinical care. Notably, we exclusively analyzed SLE patients, in contrast to prior meta-analyses that pooled several autoimmune diseases and cancer cohorts. By homing in on SLE, we excluded heterogeneity that can arise from differing disease contexts and achieved more clinically pertinent conclusions for lupus management. We also took care to exclude overlapping patient cohorts from multiple publications, thereby avoiding double-counting and ensuring each study contributed independent data. Our literature search was comprehensive up to the most recent data, and the analysis adhered to the PRISMA guidelines with a predefined protocol. We assessed study quality and bias risk. Statistical methods were appropriately chosen: we applied fixed-effect models in light of the low between-study heterogeneity, which maximizes precision when the assumption of a common effect is reasonable. Across outcomes, the consistency of findings justifies this approach and reflects the robustness of the effect. We also checked for publication bias and found no substantial asymmetry in funnel plots, increasing confidence that our results were not unduly influenced by missing negative studies. Despite the promising findings, our meta-analysis is constrained by the small number of studies and patients available, and it is mainly limited by the absence of RCTs. Although RCTs were eligible according to our inclusion criteria, no RCT met the prespecified criteria, and the quantitative synthesis was therefore based exclusively on prospective and retrospective comparative cohort studies. This represents a major limitation, as observational designs are intrinsically vulnerable to selection bias, confounding by indication, and incomplete adjustment for relevant prognostic factors, including age, baseline ovarian reserve, cumulative cyclophosphamide exposure, disease severity, and pregnancy intention, despite our best efforts to control for confounders. Consequently, although the pooled reduction in POI risk was large and highly consistent, these findings should be interpreted as strong associative evidence rather than definitive proof of causality. The small number of included studies and patients further limits the precision of estimates, particularly for pregnancy and safety outcomes. The wide confidence intervals around the pregnancy RR reflect uncertainty, and a true fertility benefit might have been missed due to insufficient sample size. Moreover, long-term reproductive outcomes were incompletely reported, and few studies provided standardized data on live birth, time to conception, ovarian reserve markers, or patient desire for pregnancy. The GRADE certainty is moderate that a GnRH-a co-therapy substantially reduces POI in SLE patients receiving cyclophosphamide, driven by a large, consistent effect in matched/adjusted cohorts. Fertility outcomes probably improve, but current evidence is of low certainty due to imprecision; larger prospective studies with standardized follow-up to live birth are needed. Safety signals are uncertain given sparse reporting, but no increase in serious AEs was detected in the available cohorts.

In conclusion, our work provides strong evidence that a GnRH agonist co-therapy is an effective and safe intervention to preserve ovarian function in women with SLE receiving cyclophosphamide. It substantially reduces the risk of POI and shows a trend toward improved fertility, without adding toxicity. Our subgroup analyses newly demonstrate that the benefit of GnRH-as is consistent across different ages and is not agent-specific, reinforcing its broad applicability. These findings fill important gaps in the literature and support current recommendations to incorporate GnRH-as into the care of reproductive-age SLE patients employing cyclophosphamide treatment. Nevertheless, given its limitations, we encourage further research, ideally multicenter prospective trials or long-term registries, to confirm these outcomes, to determine whether preserved ovarian function indeed translates into higher live birth rates, and to ensure that this fertility-preserving strategy continues to prove safe over the long term.

## Supplementary materials













## Declaration of interest

The authors declare that there is no conflict of interest that could be perceived as prejudicing the impartiality of the work reported.

## Funding

This study was funded by PIANO NAZIONALE DI RIPRESA E RESILIENZA (PNRR), MISSIONE 6 – COMPONENTE 2 – INVESTIMENTO 2.1 – VALORIZZAZIONE E POTENZIAMENTO DELLA RICERCA BIOMEDICA DEL SSN; Missione 6/componente 2/Investimento: 2.1 ‘Rafforzamento e potenziamento della ricerca biomedica del SSN’, finanziato dall’Unione europea – NextGenerationEU; and Progetto ‘PNRR-MCNT2-2023-12378364 – Early diagnosis of high-grade serous epithelial ovarian cancer through the analysis of DNA derived from Pap test smear’ (DEL. 645/2024) – CUP Istituto F93C24000330006.

## Author contribution statement

MFPM conceived the study; designed the methodology; contributed software and resources; performed validation, formal analysis, investigation, data curation, and visualization; wrote the manuscript, and administered the project. ES conceived the study; designed the methodology; performed validation; wrote, reviewed, and edited the manuscript; supervised the study; and administered the project. GC performed validation, reviewed and edited the manuscript, and supervised the study. BAM and VL reviewed and edited the manuscript and supervised the study. All authors have read and agreed to the published version of the manuscript.

## Disclaimer

The authors affiliated to the IRCCS Istituto Tumori ‘Giovanni Paolo II’, Bari, are responsible for the views expressed in this article, which do not necessarily represent the institute.
