# Supplementary material for: Gonadotropin-releasing hormone agonists during cyclophosphamide for ovarian protection in systemic lupus erythematosus
Source: Reprod Fertil. 2026 Aug 12;7(3):RAF250191. doi: 10.1530/RAF-25-0191 (PMC13539409; doi:10.1530/RAF-25-0191)
Supplement: Supplementary file 1 [file RAF-25-0191_supplementary_figure_1.pdf]

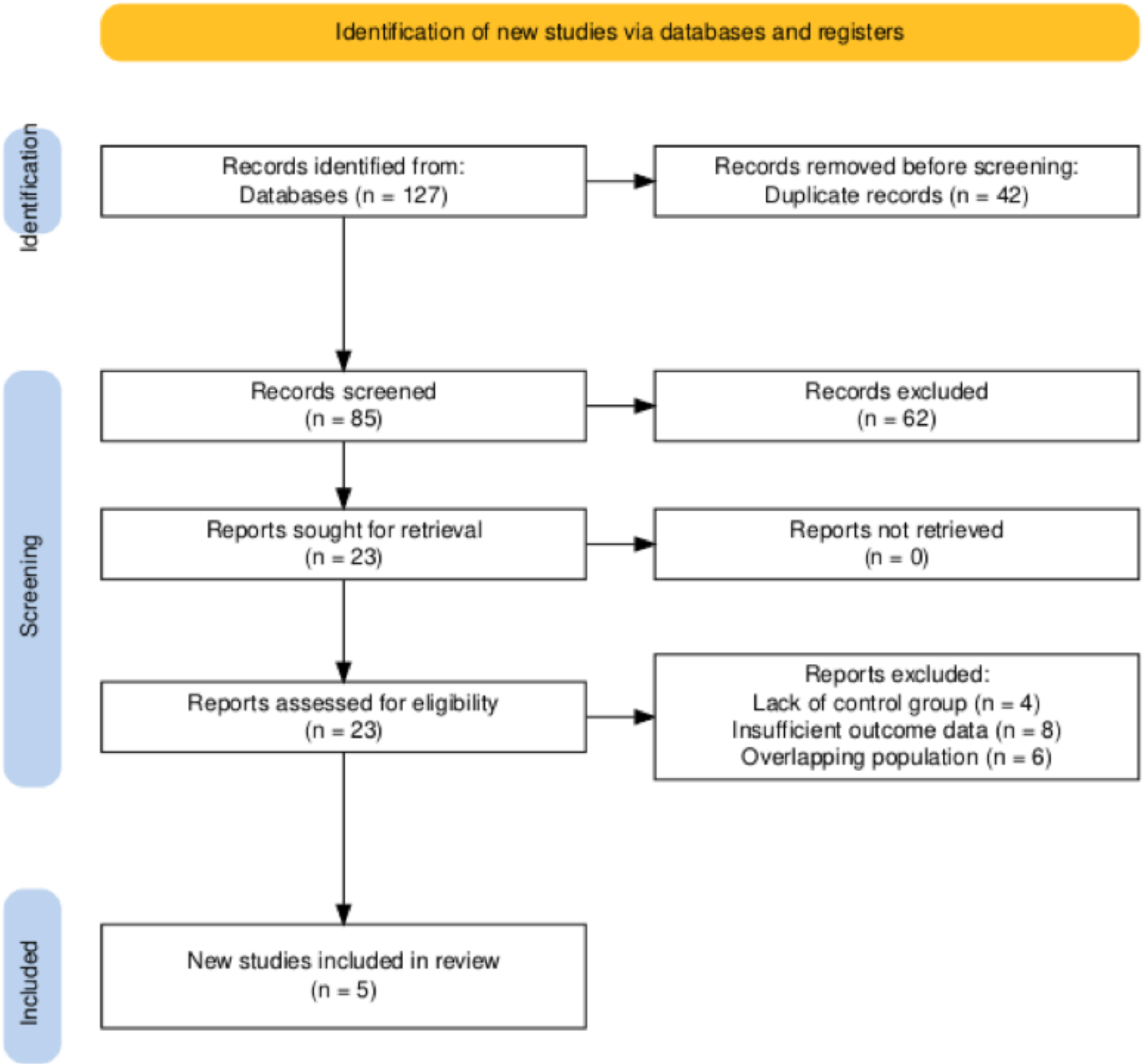

Identification of new studies via databases and registers
Identification
Records identified from:
Databases (n = 127)
Records removed before screening:
Duplicate records (n = 42)
Screening
Records screened
(n = 85)
Records excluded
(n = 62)
Reports sought for retrieval
(n = 23)
Reports not retrieved
(n = 0)
Reports assessed for eligibility
(n = 23)
Reports excluded:
Lack of control group (n = 4)
Insufficient outcome data (n = 8)
Overlapping population (n = 6)
Included
New studies included in review
(n = 5)
