# Supplementary material for: Gonadotropin-releasing hormone agonists during cyclophosphamide for ovarian protection in systemic lupus erythematosus
Source: Reprod Fertil. 2026 Aug 12;7(3):RAF250191. doi: 10.1530/RAF-25-0191 (PMC13539409; doi:10.1530/RAF-25-0191)
Supplement: Supplementary file 2 [file RAF-25-0191_supplementary_figure_2.pdf]

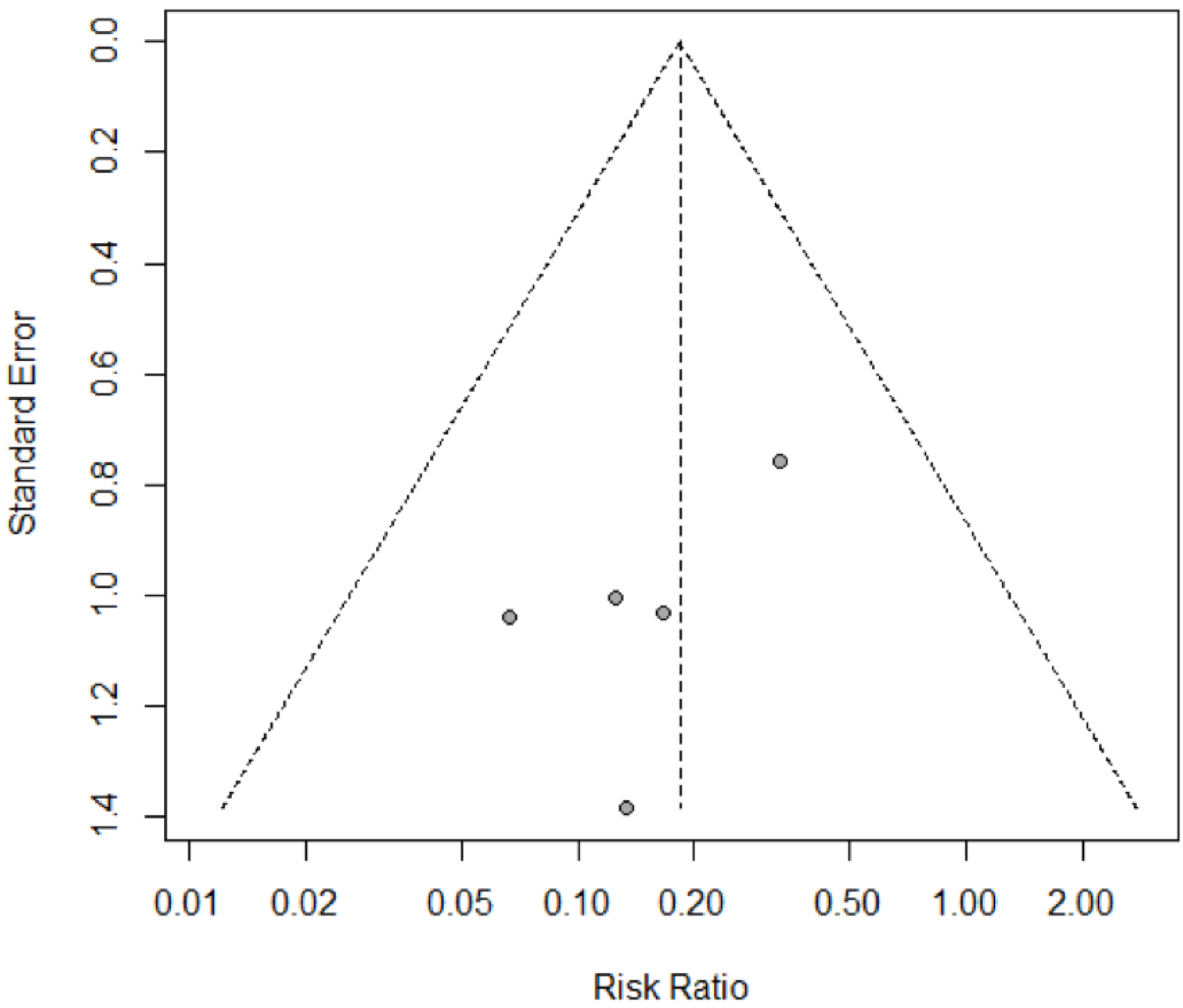
Standard Error
0.0
0.2
0.4
0.6
0.8
1.0
1.2
1.4
0.01
0.02
0.05
0.10
0.20
0.50
1.00
2.00
Risk Ratio
