# Supplementary material for: Gonadotropin-releasing hormone agonists during cyclophosphamide for ovarian protection in systemic lupus erythematosus
Source: Reprod Fertil. 2026 Aug 12;7(3):RAF250191. doi: 10.1530/RAF-25-0191 (PMC13539409; doi:10.1530/RAF-25-0191)
Supplement: Supplementary file 3 [file RAF-25-0191_supplementary_file_1.pdf]

Supplementary File 1. Search Lines for each Database screened.

PubMed (MEDLINE) final run: May 31, 2025 (filters during search: none; Humans/Female assessed at screening; date range applied in the query).

Lupus Erythematosus, Systemic[Mesh]
("systemic lupus erythematosus"[tiab] OR SLE[tiab])
1 OR 2
Cyclophosphamide[Mesh]
cyclophosphamide[tiab]
4 OR 5
Gonadotropin-Releasing Hormone[Mesh]
Leuprolide[Mesh] OR Triptorelin[Mesh] OR Goserelin[Mesh]
(("gonadotropin-releasing hormone"[tiab] OR gnrh[tiab]) AND (agonist*[tiab] OR analogue*[tiab])) OR leuprolide[tiab] OR triptorelin[tiab] OR goserelin[tiab] OR decapeptyl[tiab]
7 OR 8 OR 9
Ovarian Failure, Premature[Mesh] OR Ovarian Insufficiency, Premature[Mesh], Fertility[Mesh] OR Pregnancy[Mesh]
(premature ovarian failure[tiab] OR premature ovarian insufficiency[tiab] OR primary ovarian insufficiency[tiab] OR amenorrhea[tiab] OR ovarian reserve[tiab] OR fertility[tiab] OR pregnancy[tiab] OR gonadotoxic*[tiab])
11 OR 12
3 AND 6 AND 10 AND 13
("2000/01/01"[Date - Publication] : "2025/05/31"[Date - Publication]) AND 14

Scopus, final run: May 31, 2025 (database: Scopus; document types not restricted; date range: 2000–2025 applied in interface)

TITLE-ABS-KEY("systemic lupus erythematosus" OR SLE)
TITLE-ABS-KEY(cyclophosphamide)
TITLE-ABS-KEY(("gonadotropin-releasing hormone" OR GnRH) W/3 (agonist* OR analogue*) OR leuprolide OR triptorelin OR goserelin OR decapeptyl)
TITLE-ABS-KEY("premature ovarian failure" OR "premature ovarian insufficiency" OR amenorrhea OR "ovarian reserve" OR fertility OR pregnancy OR gonadotoxic*)
1 AND 2 AND 3 AND 4

Web of Science, final run: May 31, 2025 (indexes: SCI-EXPANDED, SSCI, ESCI; timespan: 2000–2025)

TS=("systemic lupus erythematosus" OR SLE)
TS=(cyclophosphamide)
TS=(("gonadotropin-releasing hormone" OR GnRH) NEAR/3 (agonist* OR analogue*) OR leuprolide OR triptorelin OR goserelin OR decapeptyl)
TS=("premature ovarian failure" OR "premature ovarian insufficiency" OR amenorrhea OR "ovarian reserve" OR fertility OR pregnancy OR gonadotoxic*)
#1 AND #2 AND #3 AND #4
