# Supplementary material for: Gonadotropin-releasing hormone agonists during cyclophosphamide for ovarian protection in systemic lupus erythematosus
Source: Reprod Fertil. 2026 Aug 12;7(3):RAF250191. doi: 10.1530/RAF-25-0191 (PMC13539409; doi:10.1530/RAF-25-0191)
Supplement: Supplementary file 4 [file RAF-25-0191_supplementary_table_1.pdf]

Table S1. PICOS framework.

Population (P)	Premenopausal fertile women diagnosed with SLE, undergoing IV cyclophosphamide therapy
Intervention (I)	Gonadotropin-releasing hormone agonists (GnRH-a)
Comparator (C)	Cyclophosphamide alone
Outcomes (O)	Primary: Incidence of POI or POF (historical term).<br>Secondary: pregnancy rates and reported AEs of any grade
Study Design (S)	RCTs, comparative cohort study (prospective or retrospective)

AE(s), adverse event(s); IV, intravenous; POI, premature ovarian insufficiency; POF, premature ovarian failure; RCT(s), randomized controlled trial(s); SLE, systemic lupus erithematosus
