# Supplementary material for: Gonadotropin-releasing hormone agonists during cyclophosphamide for ovarian protection in systemic lupus erythematosus
Source: Reprod Fertil. 2026 Aug 12;7(3):RAF250191. doi: 10.1530/RAF-25-0191 (PMC13539409; doi:10.1530/RAF-25-0191)
Supplement: Supplementary file 5 [file RAF-25-0191_supplementary_table_2.pdf]

Table S2. Risk of Bias assessment. The green dot represents a low risk of bias, the yellow and red dots represent a moderate and high risk of bias, respectively.

Study	Confounding	Selection of Participants	Classification of Interventions	Deviation from Intended Interventions	Missing Data	Measurement of Outcomes	Selection of the Reported Results	Overall
Blumenfeld et al. (2000)	□	□	□	□	□	□	□	□
Blumenfeld et al. (2011)	□	□	□	□	□	□	□	□
Koga et al. (2018)	□	□	□	□	□	□	□	□
Orefice et al. (2020)	□	□	□	□	□	□	□	□
Somers et al. (2005)	□	□	□	□	□	□	□	□
