# Supplementary material for: Gonadotropin-releasing hormone agonists during cyclophosphamide for ovarian protection in systemic lupus erythematosus
Source: Reprod Fertil. 2026 Aug 12;7(3):RAF250191. doi: 10.1530/RAF-25-0191 (PMC13539409; doi:10.1530/RAF-25-0191)
Supplement: Supplementary file 6 [file RAF-25-0191_supplementary_table_3.pdf]

Table S3. Summary of findings.

Outcome	No. of participants (studies)	Relative effect (95% CI)	Assumed risk (control)*	Absolute effect (per 1000)	Certainty (GRADE)	Notes
Premature ovarian insufficiency (POI)	162 (5)	RR 0.16 (0.07–0.37)	406/1000	↓ **341 fewer** (from 256 fewer to 379 fewer)	**Moderate** □[1] □[2]	Large, consistent reduction in POI across all studies.
Pregnancy (post-treatment)	147 (4)	RR 1.82 (0.86–3.86)	100/1000	↑ **82 more** (from 14 fewer to 286 more)	**Low** □[2]□[3]	Direction favors GnRH-a, but CI includes no effect; limited sample.
Serious adverse events	70 (2)	RR 0.99 (0.50–1.93)	294/1000	↑/↓ **3** (from 147 fewer to 273 more)	**Low** □[2]□[3]	No clear difference; estimates are imprecise and sparse.

*Assumed risks are pooled control risks from dataset: POI 28/69=406 per 1000; Pregnancy 6/60=100 per 1000; Serious AEs 10/34=294 per 1000.

1. **Upgrade for very large effect**: POI RR≈0.16 (CI entirely <0.5) and consistent (I^2=0%), unlikely to be fully explained by residual confounding.
2. **Risk of bias**: Nonrandomized cohorts; although matching/adjustment were used, two studies were retrospective with potential confounding by indication ⇒ **downgrade 1**.
3. **Imprecision**: CI includes no effect (pregnancy) or is wide with small total N (serious AEs) ⇒ **downgrade 1** (pregnancy) to **2** (serious AEs).
